# Text-Book of Medical Chemistry

**Published:** 1890-03

**Authors:** J. A. Miller


**Text-Book of Medical Chemistry.** For Medical and Pharmaceutical Students and Practitioners. By ELIAS H. BARTLEY, B. S., M. D., Professor of Chemistry and Toxicology, and Lecturer on Diseases of Children in Long Island College Hospital; late Chief Chemist to the Department of Health, City of Brooklyn, etc., etc. Second edition with illustrations; pp. 423. Philadelphia: P. Blakiston, Son & Co. 1890.

Medical chemistry should be a study of chemistry in its relation to normal and morbid performance of the functions of each individual organ of the body, and the relation of each organ to the entire animal economy. This subject, which is of so much importance to the physician, should be taught, not from the standpoint of the pure science, but from the vantage ground of *physiology* and *pathology*. It is greatly to be regretted that so many lecturers on the subject persist in making it merely a review of the principles of elementary chemistry, to which is added tables showing the composition of the fluids of the body and a little urinalysis. Far more advantageous would it be to the student and practitioner if it were made—

I. A consideration of chemistry in its relation to the performance of the normal functions of all the organs of the body.

II. A consideration of chemistry in its relation to any morbid declensions from the normal performance of those functions peculiar to the various organs of the existing animal.

The author carries out the old and too frequent method in the book before us. As a *Text-book* of MEDICAL CHEMISTRY, the only criticism we have to offer is, that the work is not what it is claimed to be. While this criticism may appear harsh, it is nevertheless true, for the work does not treat of the chemistry of the human body in health and disease.

In this text-book of elementary chemistry, although written in a clear and concise style, the author neglects to lead his reader, gradually, up to the important laws which form the basis upon which the science of chemistry rests.

In the study of a new science, the best interests of the student demand that he be not at once plunged into a mass of facts and a host of laws, but that he be gradually led up by means of existing analogies to the facts to be explained, and eventually to the laws of the science.

The almost immediate use of such terms as "negative radicals," etc., is extremely confusing to the student, to say the least.

That portion of the work devoted to ORGANIC CHEMISTRY, while abreast of the times, is somewhat rambling in character. The author fails to bring out the beautiful symmetry and systematic arrangement which is characteristic of this branch of the science, while the neglect to show existing analogies renders the subject an extremely difficult one for the student to grasp.

Organic chemistry, although the easiest branch of the science, is ordinarily considered the most difficult, because the subject is not presented in a logical and systematic manner.

From what has thus far been said, it must not be considered that this book is entirely destitute of good points. On the contrary, it contains much to be commended.

The explanation of the terms "Volt," "Ohm," and "Ampère," are clear and concise, and no one need confuse them after what the author has to say.

The introduction of the physiological action of the various elements upon the human system is an excellent thing in a work intended for the use of medical students and practitioners.

The classification of the elements according to the *periodic law*, is at best a difficult one, and frequently very unsatisfactory when this classification is used in the lecture-room. Prof. Bartley is to be congratulated upon the success of his arrangement according to the periodic law.

J. A. MILLER.

